# Bcl-6-directed follicular helper T cells promote vascular inflammatory injury in diabetic retinopathy

**DOI:** 10.7150/thno.43731

**Published:** 2020-03-04

**Authors:** Yan Liu, Ziqi Yang, Peilong Lai, Zijing Huang, Xiaowei Sun, Tian Zhou, Chang He, Xialin Liu

**Affiliations:** 1 State Key Laboratory of Ophthalmology, Zhongshan Ophthalmic Center, Sun Yat-Sen University, Guangzhou 510060, P. R. China; 2 Department of Hematology, Guangdong General Hospital, Guangdong Academy of Medical Sciences, Guangzhou, 510080, P.R. China.

**Keywords:** follicular helper T cells, diabetic retinopathy, Bcl-6, IL-21

## Abstract

Diabetic retinopathy (DR) is a vision-threatening complication of diabetes mellitus characterized by chronic retinal microvascular inflammation. The involvement of CD4+ T cells in retinal vascular inflammation has been considered, but the specific subset and mechanism of T cell-mediated response during the process remains unclear. Here, we aim to investigate the potential role of follicular helper T (Tfh) cells, a newly identified subset of CD4+ T cells in retinal vascular inflammation in DR.

**Methods**: Patients with DR were enrolled and the PD-1^+^CXCR5^+^CD4^+^ Tfh cells were detected in the peripheral blood by flow cytometry. The streptozotocin (STZ)-induced DR model and oxygen-induced retinopathy (OIR) model were established, and 79-6, an inhibitor of Bcl-6, was injected intraperitoneally to suppress Tfh cells. The Tfh cells-related genes were investigated in the spleen, lymph nodes, and retina of mice by flow cytometry, immunofluorescence, and qPCR.

**Results**: The Tfh cells expanded in the circulation of patients with DR and also increased in circulation, lymph nodes and retinal tissues from the STZ-induced DR mice and OIR mice. Notably, inhibition of Bcl-6, a critical transcription factor for Tfh cells development, prevented upregulation of Tfh cells and its typical IL-21 cytokine, and ameliorated vascular leakage in DR mice or retinal angiogenesis in OIR mice, indicating that Bcl-6-directed Tfh cells could promote vascular inflammation and angiogenesis.

**Conclusions**: Our results suggested that excessive Bcl-6-directed Tfh cells represent an unrecognized feature of DR and be responsible for the retinal vascular inflammation and angiogenesis, providing opportunities for new therapeutic approaches to DR.

## Introduction

Diabetic retinopathy (DR) is the most common microvascular complication of diabetes and the main cause of blindness in the working-age population 1-3. Up to 80 percent of people who have diabetes for 20 years or more would suffer from DR 4. The longer a person has diabetes, the more chance he/she develops DR despite with appropriate glucose control. Recently, a chronic low-grade inflammation has been recognized as a characteristic immunopathologic change in DR 5-7. The hallmark of inflammation-associated events during DR include upregulation of inflammatory mediators and trafficking and activation of various immune cells, in particular CD4+ T cells 8, 9.

CD4+ T cells are generally recruited to the vessel wall in conjunction with macrophages, orchestrating the inflammation and accelerating vascular injuries 10. In the patients with DR, accumulation of CD4+ T cells, B cells, and macrophages is observed in their vitreous 9, 11, 12. Some studies reported advanced glycation end products would promote CD4+ T cells differentiation toward pro-inflammatory state 13, whereas regulatory T cells exhibit reversing role on insulin resistance in Type 2 diabetes 14, indicating that the dysregulation of CD4+ T cells was implicated in the inflammatory response during diabetes. However, the specific type of CD4+ T cell and its role in DR are poorly characterized.

Conventional CD4+ effector T cells include Th1, Th2, and Th17 cells. Recently, a new subset namely follicular helper T (Tfh) cells, have attracted close attention for their role in vaccine-elicited immune responses, protective immunity in malignancy and diverse biological processes 15, 16. Tfh cells, most commonly identified as PD-1^+^CXCR5^+^CD4^+^ cells, initially contribute to development of B cells in Germinal Center (GC) 17. Beyond its restricted role in GCs, Tfh cells that reside in extrafollicular areas may also promote diseases independently of helping antibody responses 15, 17. Remarkably, several studies have reported that patients with diabetes presented elevation of CD4+ T cells with a Tfh phenotype in the peripheral blood 18, 19. However, it is still unclear that whether Tfh cells accelerate local tissue inflammation and induce complication of diabetes, which is highly warranted for clarifying the pathological mechanism. In the context of DR, our study aims to find whether aberrantly regulated Tfh would migrate into retina and play an important role in vascular inflammatory injuries.

Here, our results firstly showed that circulating Tfh cells were overrepresented in DR patients. We further explored its role in the streptozotocin (STZ)-induced DR mice and retinal angiogenesis model of oxygen induce retinopathy (OIR) mice. The data provided evidence that Bcl-6 directed Tfh cells played an important role in the inflammatory process during DR.

## Results

### PD-1^+^CXCR5^+^CD4^+^ Tfh cells were increased in the circulation of DR patients and STZ-induced DR mice

**Figure 1:**
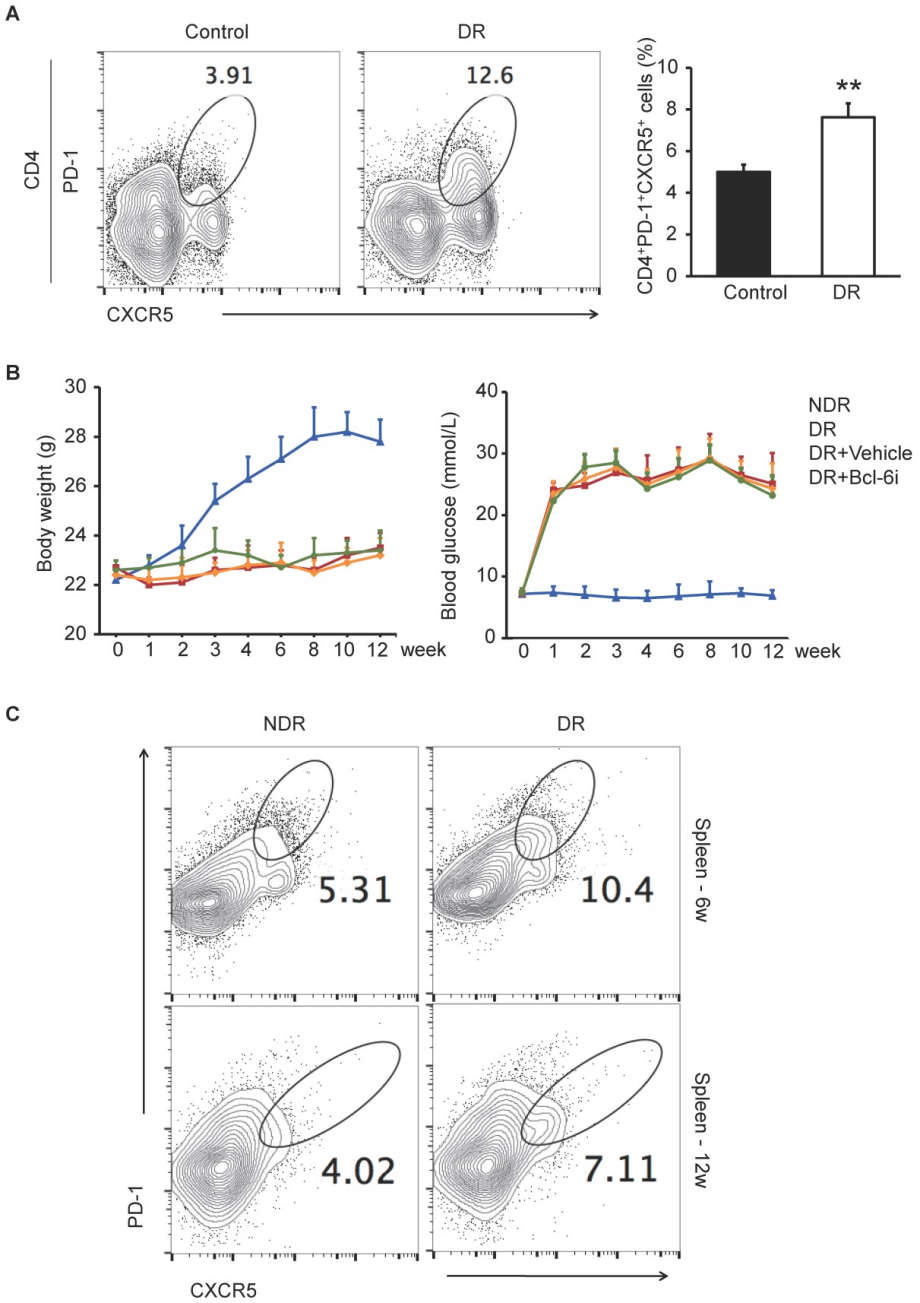
The excessive PD-1^+^CXCR5^+^CD4^+^ Tfh cells in DR patients and STZ-induced DR mice.** (A)** Flow cytometry analysis showed the percentage of PD-1^+^CXCR5^+^ cells in CD4+ T subset in peripheral blood was significantly higher in DR patients compared with age-matched non-diabetic control. **P<0.01. **(B)** The line chart showed the changes of body weight and blood glucose of STZ-induced DR mice and controls. Bcl-6i, Bcl-6 inhibitor. **(C)** Flow cytometry analysis showed that both 6 weeks and 12 weeks after last STZ injection, the percentage of PD-1^+^CXCR5^+^CD4^+^ Tfh cells in spleen was significantly higher in DR mice compared with age-matched normal ones.

It has been reported that CXCR5^+^CD4^+^ T cells in peripheral blood are the circulating counterparts of GC Tfh cells in secondary lymphoid tissue and represent recently activated cells 20, 21. To identify cells with a Tfh-cell phenotype in DR, we first detected CD4+ T cells that were PD-1^+^CXCR5^+^ in peripheral blood from ten DR patients and ten non-diabetic controls by flow cytometry. The enrolled patients suffered from DR complication 4.8 ± 2.7 years after type 1 or 2 diabetes mellitus diagnose (The characteristics of patients are showed in Supplementary Table S1), who were very likely undergoing chronic inflammation. As shown in Figure 1A, the percentage of PD-1^+^CXCR5^+^CD4^+^ cells were significantly higher in peripheral blood samples from DR patients compared with those from non-diabetic controls.

Next, we established the DR mouse model by STZ injection and investigated the frequency of GC Tfh cells in splenocytes. STZ-induced diabetic mouse is a widely used and easily accessible animal model of diabetes. Although these mice suffered from diabetes due to insulin deficiency, which were different from the type 2 diabetic models of ob/ob or diet-induced obese mice, the STZ-induced mice presented with classical retinal vascular changes in DR such as leukostasis and vascular leakage 22. The PD-1^+^CXCR5^+^CD4^+^ GC Tfh cells were expanded markedly both 6 weeks and 12 weeks after STZ injection in the spleen of DR mice (Figure 1C), indicating that excess generation and maintaining of Tfh cells were accompanied with the progression of diabetic retinopathy.

**Figure 2:**
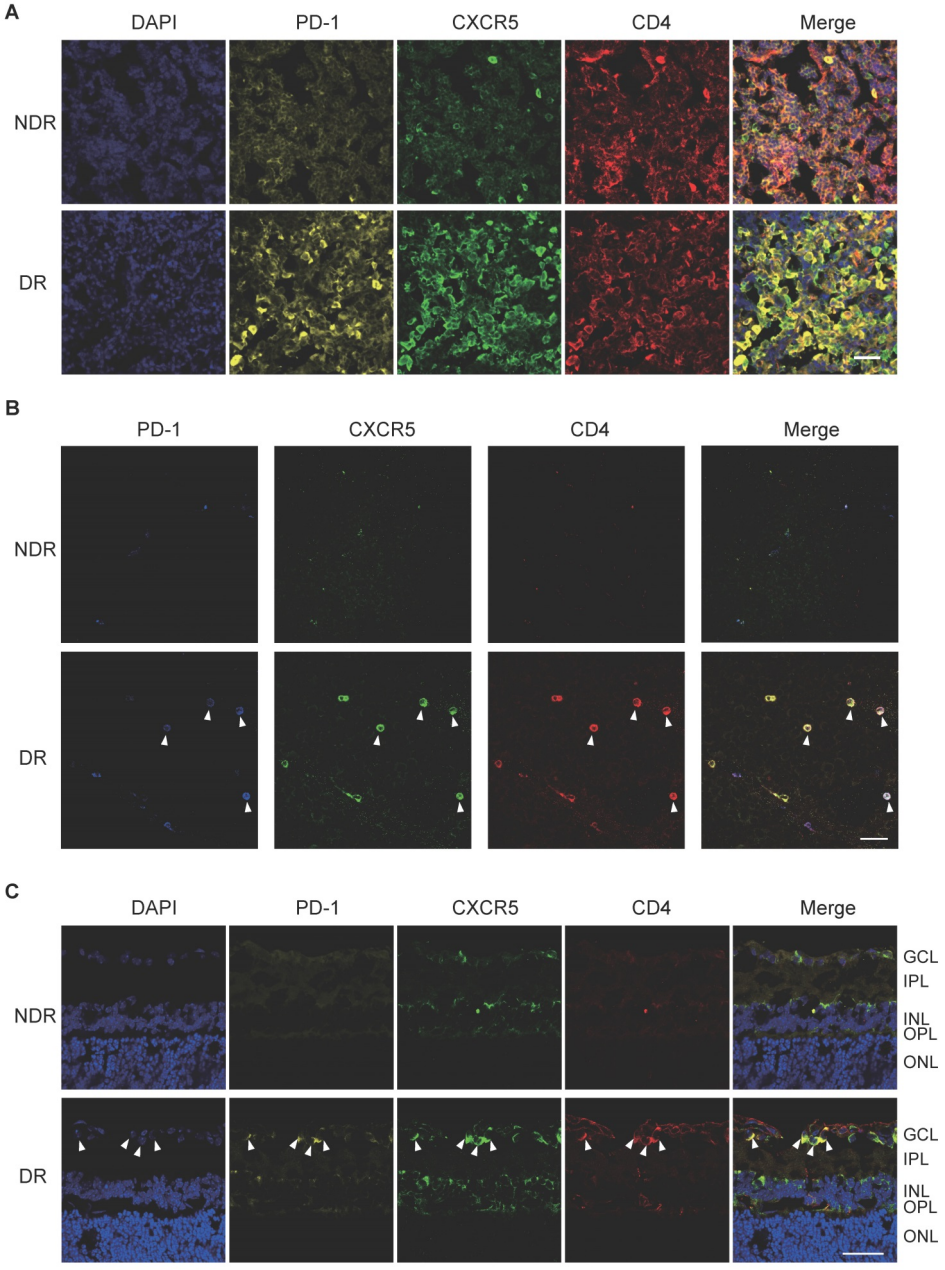
The frequency of Tfh cells was enhanced during the development of DR in STZ-induced mice. Representative images of immunofluorescence staining in cervical lateral lymph node cryosection **(A)**, retinal flat-mount **(B)**, and cryosection **(C)** showed wide distribution of PD-1^+^CXCR5^+^CD4^+^ Tfh cells in DR mouse, whereas lack of PD-1 and CXCR5 expressions on CD4+ T cells in LN** (A)** and few PD-1^+^CXCR5^+^CD4^+^ Tfh were observed in the retina from non-diabetic mice **(B-C)**. GCL: ganglion cell layer; IPL: inner plexiform layer; INL: Inner nuclear layer; OPL: outer plexiform layer; ONL: outer nuclear layer; Scale bar, 50 µm.

In addition, cervical lateral lymph nodes (LNs), the secondary lymphoid tissue for ocular lymphatic drainage, were also collected to detect the presence of Tfh cells. As shown in Figure 2A, strong co-staining of PD-1, CXCR5, and CD4 was observed obviously in the cervical lateral LNs from DR mice, though the staining of PD-1 and CXCR5 was quite weak on CD4+ T cells in the NDR control mice. Consistently, the retinal flat-mounts showed there were scarcely any Tfh cells infiltration into the retina under normal physiological condition (Figure 2B). However, at the setting of diabetic retinopathy, many PD-1^+^CXCR5^+^ CD4^+^ Tfh cells with round shape accumulated in the diabetic retina (Figure 2B). To further investigate the location of these infiltrated Tfh cells in DR, we also performed staining in retinal cryosections (Figure 2C). Obviously, most recruited Tfh cells were distributed along the ganglion cell layer (Figure 2C). These data indicated the involvement of Tfh cells in the process of diabetic retinopathy.

### Tfh cells were also upregulated in oxygen-induced retinal angiogenesis mice

**Figure 3:**
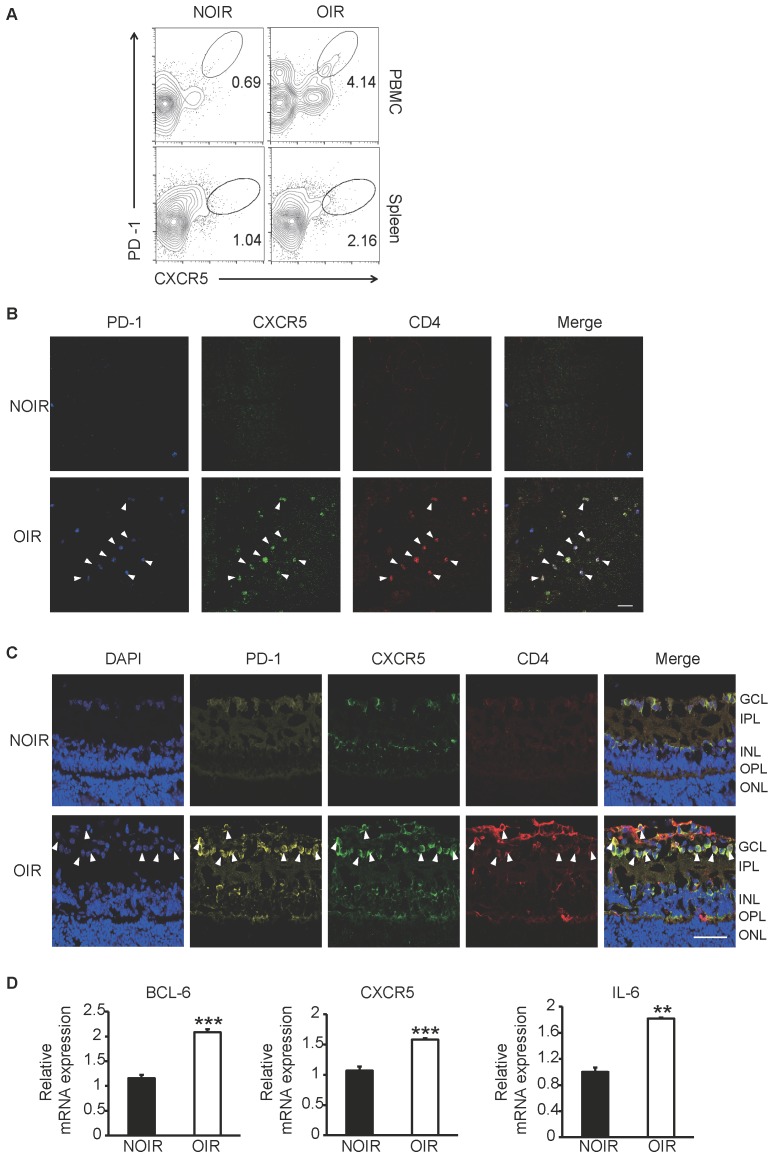
Tfh cell frequency was increased in retinal angiogenesis model of OIR.** (A)** Flow cytometry analysis showed both percentages of PD-1^+^CXCR5^+^ cells in CD4^+^ T subset in PBMC and spleen were elevated markedly in OIR mouse compared with controls at P17, the peak time of retinal angiogenesis. **(B-C)** Representative immunofluorescence staining images of retinal flat-mount and cryosection showed PD-1^+^CXCR5^+^CD4^+^ Tfh cells infiltrated into retina especially in the superficial layer during angiogenesis in OIR mouse. Scale bar, 50 μm. **(D)** Real time PCR analysis revealed the up-regulation of Bcl-6, CXCR5 and IL-21 expressions in OIR retina. **P<0.01, ***P<0.001.

Since the STZ-induced DR model was not tending to generate retinal angiogenesis 23, the OIR model, a widely used animal model for hypoxia- induced retinal angiogenesis 24, was also utilized in this study. Circulating and GC Tfh cells were detected and analyzed by flow cytometry. At the peak of retinal angiogenesis at postnatal day 17 (P17), the percentages of PD-1^+^CXCR5^+^CD4^+^ cells in blood and spleen from OIR mice increased significantly compared to those from same-age normal NOIR controls (Figure 3A). Strikingly, the CD4+ T cells highly infiltrated into the retina of OIR mice (Figure 3B-C), indicating the presence of T cells-response in the inflammatory retina during angiogenesis. Of note, many of these CD4+ T cells also expressed PD-1 and CXCR5 and mostly distributed in the superficial retinal layer, where the angiogenesis occurred (Figure 3B-C). The expression of Tfh cells-relative genes was also detected in the retina. The qPCR data showed that the mRNA expression of Bcl-6, a critical transcription factor required for programming of Tfh cells generation, was elevated in OIR retinae (Figure 3D). In addition, CXCR5 and IL-21 were also up- regulated in retinae from OIR mice (Figure 3D). Taken together, these results suggested the requirement of Tfh cells infiltration in ischemia-induced angiogenesis.

### Blockade of Bcl-6 suppressed the generation of Tfh cells and attenuated the vascular leakage during DR

**Figure 4:**
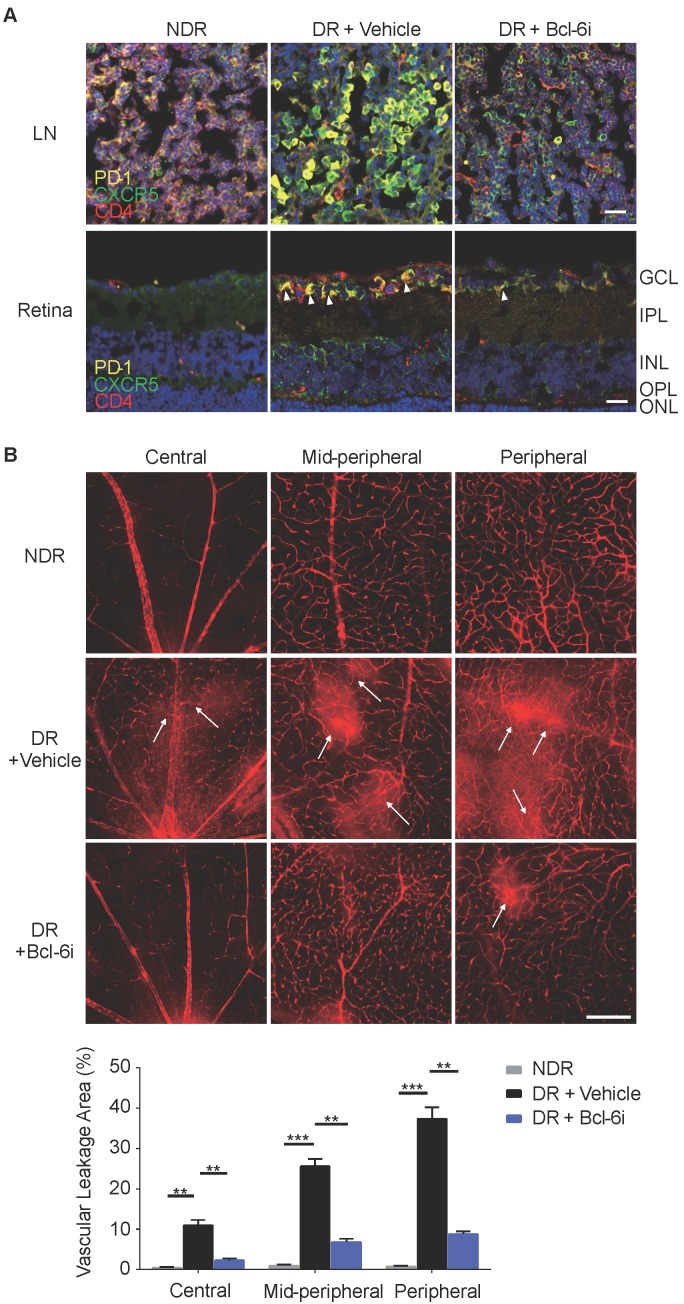
Blockade of Bcl-6 down-regulated Tfh cells and attenuated the vascular leakage during DR. **(A)** The immunofluorescence staining results revealed evident high expressions of PD-1 and CXCR5 on CD4^+^ T cells in the LN from vehicle- injected DR mice but reduced PD-1^+^CXCR5^+^CD4^+^ Tfh cells were observed after Bcl-6i injection. Similarly, PD-1^+^CXCR5^+^CD4^+^ Tfh cells were accumulated in the retinal superficial layer of vehicle-injected DR mice, whereas elevation of these cells was abolished after Bcl-6i 79-6 injection. Scale bar, 25µm. **(B)** In the normal mice, retinal vessels were sharply outlined with the Evans blue dye retained within the vessel lumen, whereas breakdown of the BRB was observed in vehicle-injected DR control mice with leakage of the Evans blue dye into the retinal parenchyma (white arrows). Treatment with Bcl-6i 79-6 induced a significant decrease in dye leakage, with a very distinct outline of the vessels. The statistics confirmed the marked reduction of vascular leakage after 79-6 injection. Scale bar, 100µm.

Bcl-6 orchestrates Tfh cell differentiation and is recognized as a critical regulator for Tfh cells generation and maintaining 25. To further evaluate the role of Tfh cells in DR, Tfh generation was blocked by 79-6, a Bcl-6 specific inhibitor (Bcl-6i), in the STZ- induced mice. As expected, 79-6 injection significantly abolished the up-regulation of PD-1^+^CXCR5^+^CD4^+^ Tfh cells in both lymph nodes and retina from DR mice compared to vehicle-injected group (Figure 4A). Further, BRB breakdown, one of the major pathologic manifestations of DR, was evaluated using Evans blue dye leakage assay. Retinal flat-mounts staining showed that obvious diffuse of the Evans blue dye from retinal vasculature in vehicle-injected diabetic mice, prominently in the mid-peripheral and peripheral locations of retina (Figure 4B). However, BRB breakdown was reduced by Bcl-6 inhibitor, as evidenced by the decrease of dye leakage into the Bcl-6i-treated retinal parenchyma (Figure 4B). These findings indicated Tfh promotes pathological breakdown of BRB in DR.

### Downregulation of Tfh cells ameliorated the retinal neovascularization

**Figure 5:**
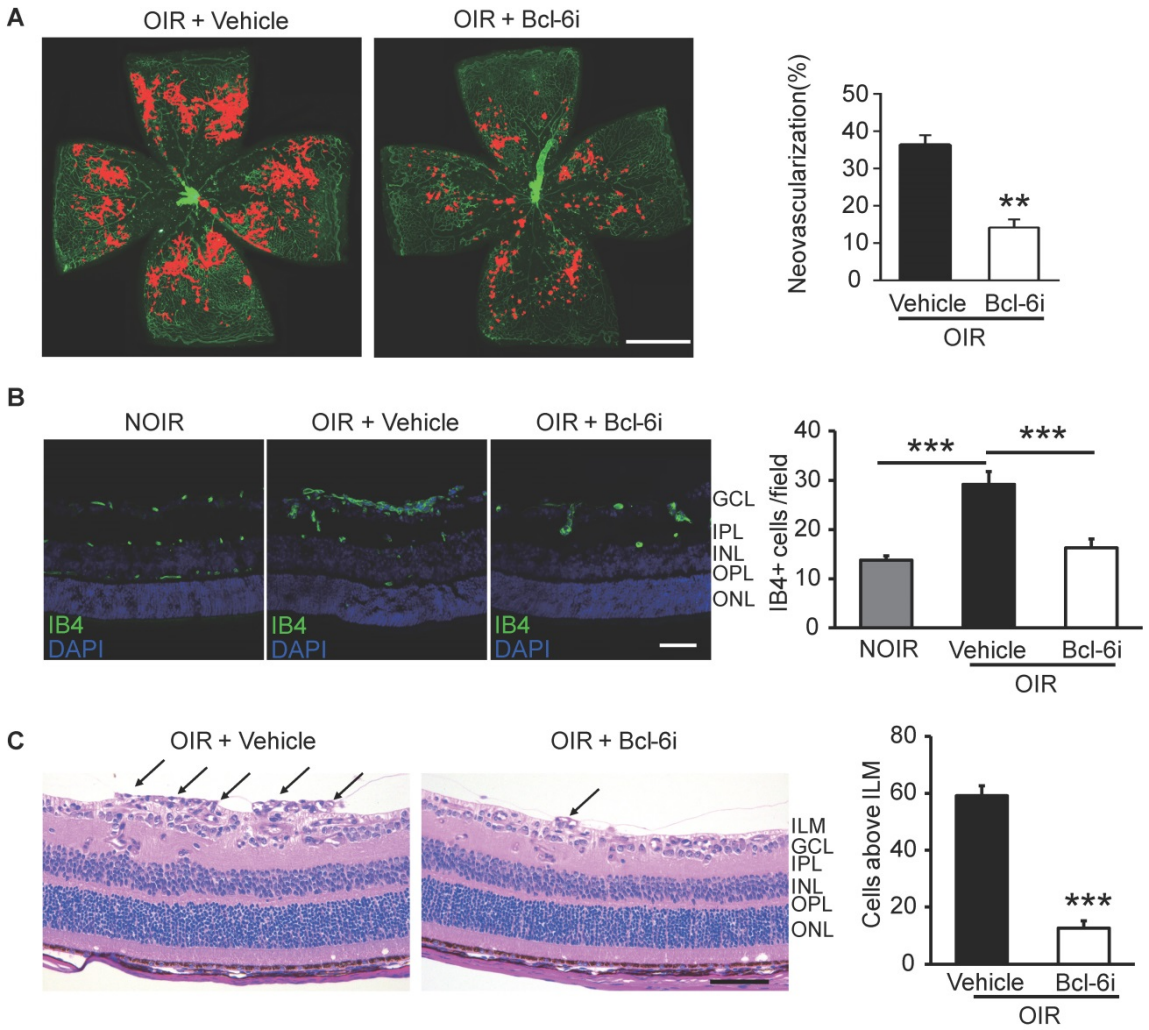
Inhibition of Tfh cells suppressed the retinal neovascularization in OIR. **(A)** Retinal flat-mounts showed that the neovascular tufts were developed markedly in the OIR retina at P17, however, the neovascularization area was significantly reduced after Bcl-6i treatment. Scale bar: 1 mm. **(B)** Immunofluorescence staining on retinal cryosections presented reduced IB4+ vascular endothelial cells in the BCL-6i-treated group, compared to vehicle-treated controls. scale bar: 50 μm. **(C)** H&E staining revealed less neovascular cells anterior to the ILM (black arrows) after BCL-6i treatment. **P<0.01, ***P<0.001. Scale bar:100 µm.

The Bcl-6i was also utilized to investigate the role of Tfh cells in retinal angiogenesis in OIR mice. The neovascularization was assessed by Isolectin B4 (IB4) staining on the superficial retina, where the ischemia-induced angiogenesis occurred remarkably. As shown in Figure 5A, vehicle-injected OIR presented with masses of irregular vascular tufts on the boundary of avascular and vascular areas, whereas Bcl-6i treatment greatly attenuated the neovascular tufts. The IB4 staining on cryosection was performed to assess the vertical profile of retinal neovascularization (Figure 5B). The data exhibited IB4+ endothelial cells proliferated markedly in the superficial layer of vehicle-injected OIR retina and much less IB4+ cells were found in the Bcl-6i group (Figure 5B). In addition, H&E staining on retinal sections was performed to characterize neovascular cells anterior to the internal limiting membrane (ILM), which is also a hallmark of angiogenesis in the OIR model. As shown in Figure 5C, the number of neovascular cell nuclei anterior to the ILM significantly decreased in the Bcl-6i-treated group, implicating an extraordinary anti-angiogenesis effect of Bcl-6 blockade.

### Tfh cells promoted the production of IL-21 and pro-angiogenesis cytokines in STZ-induced DR mice and OIR mice

**Figure 6:**
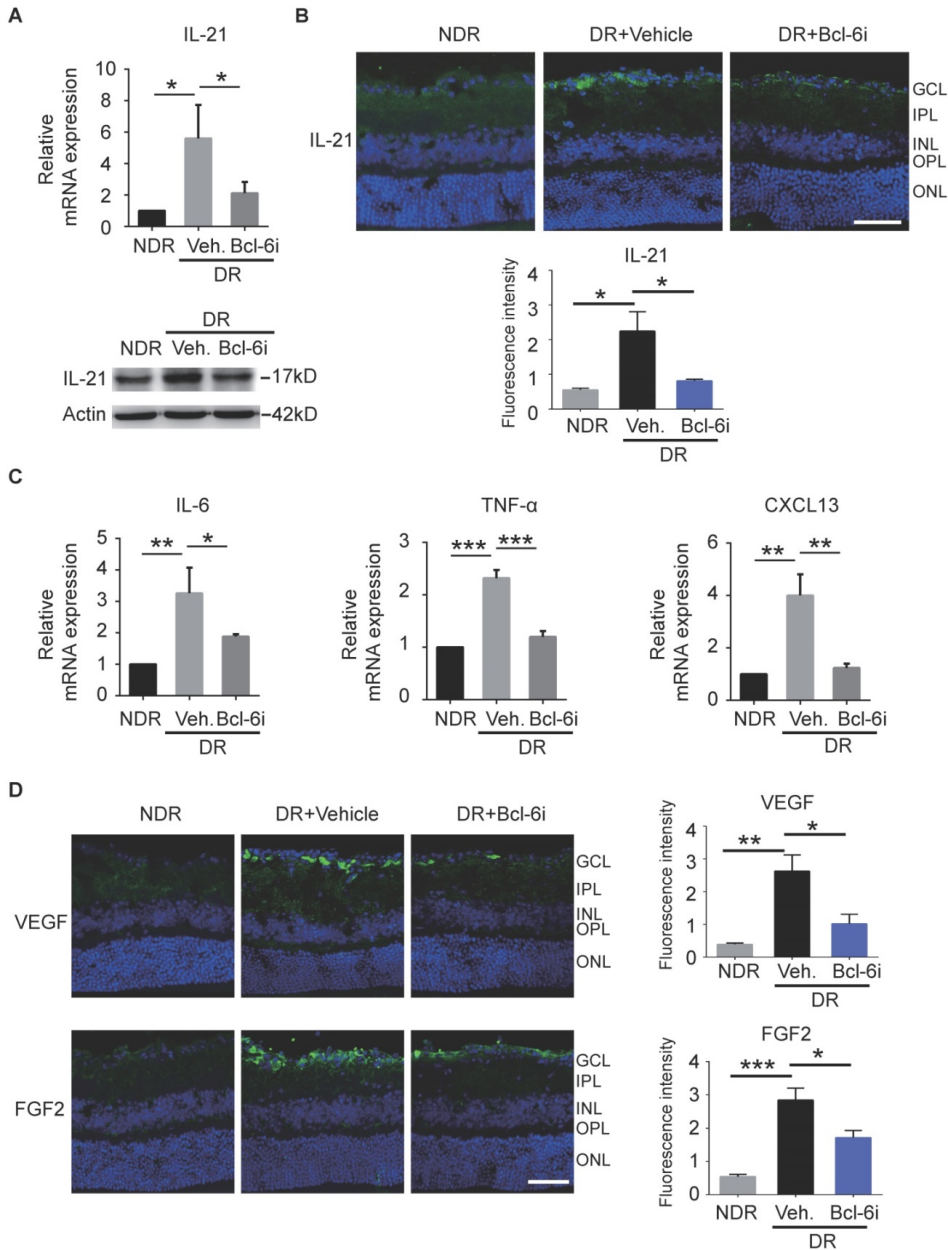
The generation of IL-21 and some pro-angiogenesis factors was decreased after Bcl-6 inhibitor treatment. **(A)** qPCR and WB analysis showed both the RNA and protein levels of IL-21 were dramatically up-regulated in the retinae from vehicle-injected DR mice, which was abrogated by Bcl-6i administration.** (B)** The immunofluorescence staining revealed that IL-21 was distributed mainly in the superficial layers of DR mice, while absence of IL-21 was observed in the NDR mice. However, Bcl-6i treatment inhibited the expression of IL-21 in the DR mice. Scale bar: 50 μm. **(C)** qPCR results showed mRNA expressions of IL-6, TNF-α and CXCL13 were increased in DR mouse, whereas BCL-6i administration suppressed their expressions. **(D)** Representative images of retinal cryosections showed strong staining of VEGF and FGF2 in vehicle-injected DR mice, whereas significantly reduced staining was observed after BCL-6i administration. Scale bar: 50 μm. *P<0.05, **P<0.01, ***P<0.001.

Recent studies reported Tfh cells provide help to B cells in an IL-21-dependent fashion 17. Receptor for IL-21 is expressed on endothelial cells and IL-21 could improve ECs proliferation and tube formation *in vitro* 26. It is therefore speculated that the contribution of Tfh cells in DR may depend on IL-21 cytokine. As expected, both mRNA and protein levels of IL-21 cytokine were increased in retinae from vehicle-injected DR mice, whereas Bcl-6 inhibitor abolished the effect (Figure 6A). The immunostaining data showed that IL-21 was distributed prominently at the superficial layer, where the vascular leakage mostly occurred (Figure 6B). In addition, the elevation of IL-21 production was abrogated by Bcl-6i treatment (Figure 6B). Other pro-inflammatory cytokines were also detected and qPCR data showed that IL-6 and TNF-α were also increased in the vehicle-injected DR retina and decreased after Bcl-6i treatment. CXCL13 chemokine, a typical ligand of CXCR5 expressed on Tfh cells, was up-regulated in the vehicle-injected DR retina, implicating the potential role of CXCL13/ CXCR5 signaling on Tfh recruitment (Figure 6C). Since VEGF and FGF2 have been considered as two critical pro-angiogenesis cytokines in DR, we also detected their expressions and found both VEGF and FGF2 were highly expressed in the vehicle-injected DR retinae, whereas Bcl-6i treatment reduced them (Figure 6D), suggesting that Tfh cells accelerated DR possibly through inducing pro-angiogenesis cytokine expressions.

**Figure 7:**
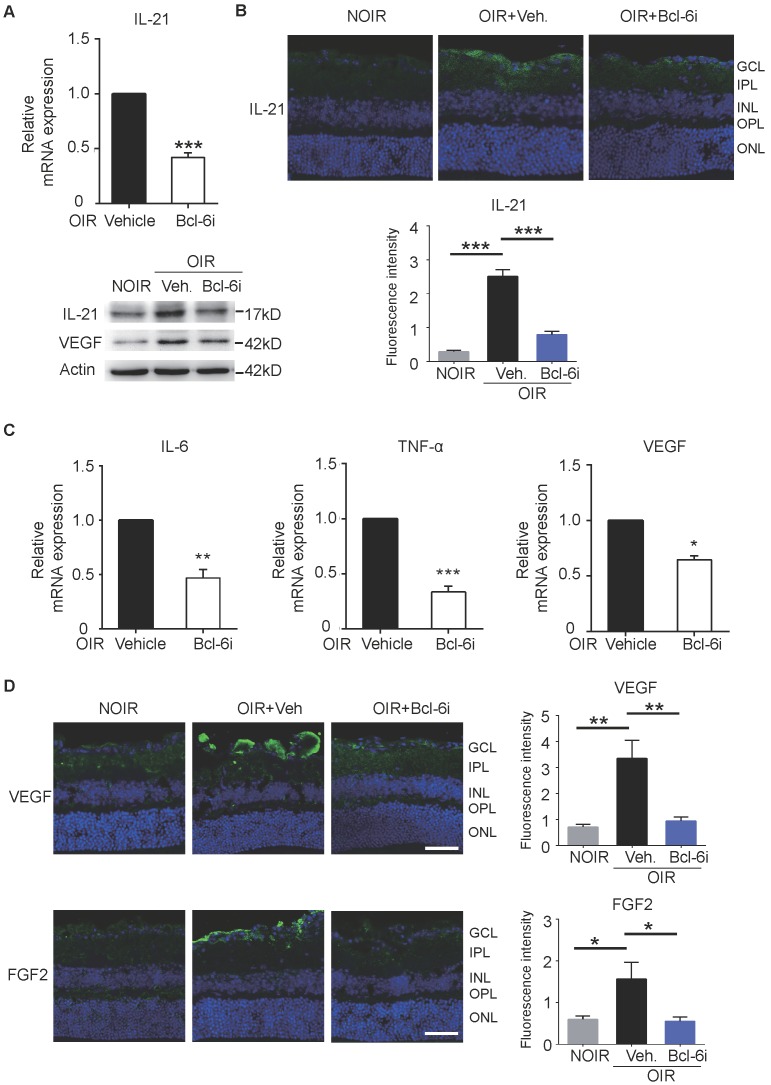
Blockade of Bcl-6 suppressed the expression of IL-21 in OIR retinae.** (A)** Bcl-6i administration significantly suppressed IL-21 and VEGF expression in OIR mice. ***P<0.001** (B)** The immunofluorescence staining revealed that IL-21 was distributed mainly in the superficial layers of OIR mice, while absence of IL-21 was observed in the NOIR mice. However, Bcl-6i treatment inhibited the expression of IL-21 in the OIR mice. Scale bar: 50 μm.** (C)** BCL-6i administration suppressed mRNA expression of IL-6, TNF-α and VEGF in OIR mice compared with vehicle-treated control. **(D)** It was weak staining of VEGF and FGF2 in the normal NOIR retinae, whereas evident VEGF and FGF2 distribution along the superficial layer in vehicle-treated OIR retinae, where the neovascularization occurred markedly. However, there were reduced expressions of VEGF and FGF2 in the BCL-6i-treated OIR retinae. *P<0.05, **P<0.01, Scale bar: 50 µm.

In order to assess the mechanism of Tfh cells-mediated retinal angiogenesis in OIR mice, Bcl-6 inhibitor was also injected in these mice to block Tfh cells. Consistent with the data in DR mice, IL-21 in retina was increased in vehicle-treated OIR and down-regulated by Bcl-6i injection (Figure 7A, B). Bcl-6i-treated group exhibited lower levels of pro-inflammatory cytokines (IL-6 and TNF-α) and less production of pro-angiogenesis cytokines (VEGF and FGF2) compared with vehicle-treated OIR mice (Figure 7C-D). Collectively, these data demonstrated that Tfh cells produced IL-21 cytokine, as well as other pro-inflammatory and pro-angiogenic cytokines in diabetic retinopathy and retinal neovascularization *in vivo*.

## Discussion

In this study, Tfh cells were firstly identified to contribute to the pathogenesis of diabetic retinopathy. Tfh cells are a recently identified subset of CD4+ T cells and uncontrolled generation of Tfh cells might lead to autoimmunity and inflammation 17, 27.

We provided evidence showing that circulating Tfh cells were censored in normal subjects, but significantly expanded in patients with DR. The excessive Tfh cells were also observed in the circulation, secondary lymph nodes and retinae from DR and OIR mice, which may represent a new feature of diabetic retinopathy. Of note, Tfh cells attenuation by Bcl-6 inhibitor 79-6 exhibited a remarkable effect on suppressing the pathologic retinal vascular leakage and angiogenesis *in vivo*, further implicating that Tfh cells may be responsible for the retinal inflammation and angiogenesis in DR.

Besides GCs in lymphoid tissues, Tfh cells were also identified in nonlymphoid tissues, expanding their involvement in inflammatory diseases 15. It is increasingly recognized that ectopic lymphoid structure frequently form at sites of chronic inflammation in non-lymphoid organs in response to infection, auto‐immunity, or environmental irritants 28, 29. The circulating Tfh cells would migrate to the sites of ectopic GCs formation. In this study, CXCL13, a typical chemokine for recognizing and recruiting CXCR5+ cells 15, was elevated in retina at the setting of hyperglycemia, probably facilitating migration of Tfh cells into diabetic retinae. In addition, we also found a higher level of TNF-α in DR retinae, accompanied with infiltration of Tfh cells into retina. Actually, TNF-α exhibited a well-established role in lymphoid neogenesis partly through CXCL13. It was proposed that TNF-α induced generation of CXCL13 in endotheliums and synoviocytes from rheumatoid arthritis mice, leading to recruitment of Tfh cells and development of ectopic GCs 30. Therefore, it was speculated that TNF-α and CXCL13 were involved in recruitment of Tfh cells and establishment of inflammatory condition in DR.

The circulating Tfh cells shared nearly same phenotypes and functions with GC Tfh cells 20. In consistent with previous studies, we found in this study Tfh cells with PD-1^+^CXCR5^+^CD4^+^ phenotype exhibited similar change of frequency in blood, spleen, lymph nodes, and retina in DR mice, indicating the migration and shuffle of Tfh cells. Actually, Tfh cells could shuffle between peripheral blood and lymphoid tissues, facilitating the detection of Tfh cells in humans 31, 32. Thus, it is reasonable to conclude that the increase of PD-1^+^CXCR5^+^CD4^+^ Tfh cells in peripheral blood from DR patients could reflect GCs dysregulation and aberrant immune responses in the lymphoid and non-lymphoid tissues during diabetes.

Tfh cells promote chronic vascular inflammation in DR possibly through multiple mechanisms. Firstly, Tfh cells themselves could improve other immune cells recruitment and facilitate or maintain ectopic follicles structures and functions, helping to shape the character and magnitude of the inflammatory response in tissues 15. In our study, it is highly possible that infiltration of Tfh cells in diabetic retinae shaped the proinflammatory cytokine profile, sustaining a local inflammatory microenvironment that could nurture for other immune cells with more direct pathogenic roles. Secondly, IL-21, the typical effector cytokine of Tfh cells, could in turn improve the generation and differentiation of Tfh cells, amplifying the pathogenic role of Tfh cells 33, 34. Moreover, IL-21 was reported to increase the viability of vascular endothelial cells which express the receptor of IL-21, highlighting the potent pro-angiogenic role of IL-21 26. In this study, large amounts of IL-21 were found in retina from DR and OIR mice, indicating the potential effect of IL-21 in retinal angiogenesis. It would be interesting to further explore the mechanism of angiogenesis specifically to IL-21. Finally, other pro-angiogenic growth factors such as VEGF and FGF2 were also elevated in DR retinae and decreased after Tfh cells blockade, further indicating the pro-angiogenic role of Tfh cells in DR.

Notably, Bcl-6 is the defining transcription factor of Tfh cells and auto-regulates its own expression, dominantly directing T cells toward Tfh cells and away other Th lineage cells 35. Although Bcl-6 regulated directly just a few genes in Tfh cells, accumulating evidences suggested that Bcl-6 orchestrated Tfh cells generation and differentiation via multiple distinct mechanisms 35, 36. Bcl-6 exhibited broad effects on Tfh biology and function and Bcl-6 inhibitor could be utilized to attenuate Tfh generation and function in experiments. In this study, we used 79-6, which was a commercial Bcl-6 inhibitor and non-toxic to animals with favorable pharmacokinetics 37, to suppress the generation of Tfh cells. Although the 79-6 efficiency for function loss of Tfh cells needs to be improved, it is now a widely used inhibitor for Bcl-6. In this study, we found that Bcl-6 targeting with the small molecule 79-6 resulted in not only the reduction of Tfh cells in LNs and retina, but also less production of IL-21 and other pro-inflammatory cytokines in retina, underlying the fact that Bcl-6 was closely associated with the fate of Tfh cells and 79-6 represent an effective anti- angiogenesis strategy *in vivo.*

In summary, our data demonstrated for the first time that dysregulated Tfh cells, a new type of CD4+ T cells, contribute to retinal vascular inflammation in DR. Tfh cells blockade by Bcl-6 inhibitor suppressed the vascular leakage and retinal angiogenesis, accompanied with less production of IL-21 and other pro-inflammatory and pro-angiogenic cytokines. The results provide promise for elucidating the mechanism of CD4+T cell-mediated vascular inflammation in DR and propose that targeting Tfh cells may be an attractive rationale for treating DR.

## Materials and Methods

### Ethics approval and consent to participants

The study was conducted at Zhongshan Ophthalmic Center, Sun Yat-sen University. The protocol was approved by the Ethics Committee of Zhongshan Ophthalmic Center at the Sun Yat-sen University in accordance with the tenets of the Declaration of Helsinki and informed consents from patients and healthy subjects were obtained. The animal studies conformed to the guidelines for care and use of laboratory animals in accordance with the ARVO Statement for the Use of Animals in Ophthalmic and Vision Research, as well as the Institutional Animal Care and Use Committee of Zhongshan Ophthalmic Center, Sun-Yat Sen University. C57BL/6J mice were purchased from Guangzhou University of Chinese Medicine and housed in Zhongshan Ophthalmic Center with a specific pathogen-free and temperature-controlled facility on a 12 h light/dark cycle. Mice were sacrificed by carbon dioxide euthanasia and samples (spleen, lymph node, blood and retina) were collected.

### Characteristics of participants

A total of 20 participants were recruited with 10 DR patients (5 females, 5 males, 56.8 ± 7.3 years) and 10 healthy subjects (6 females, 4 males, 58.6 ± 8.9 years). The characteristics of DR patients were listed in Supplementary Table S1. All recruited participants have signed the informed consent. Ten microliter blood from each participant was collected by heparin sodium anticoagulant tubes for flow cytometry analysis.

### Diabetic model and oxygen-induced retinopathy model

For the induction of diabetes, 6-week-old male C57BL/6J mice were fasted overnight and then injected intraperitoneally with streptozotocin (STZ, 60 mg/kg, Sigma, St. Louis, MO) for 5 consecutive days. STZ was dissolved in cold 100 mM pH4.5 citrate buffer and prepared freshly for immediate use. The blood glucose levels in samples obtained from the tail vein were measured one week after the final injection and then once per week. Only mice with blood glucose level > 300 mg/dL (16.6 mmol/L) were considered diabetic and further used in the study. Age-matched male mice were injected intraperitoneally with equal citrate buffer as NDR controls. As shown in Figure 1B, the body weight of STZ-induced diabetic mice was much lower than that of non-diabetic mice and the blood glucose maintained above 16.6 mmol/L in the diabetic mice. For treatment, the diabetic mice were injected intraperitoneally with Bcl-6 inhibitor (Bcl-6i) 79-6 (50 mg/kg, Merck Millipore, Darmstadt) dissolved in 10% Dimethyl Sulfoxide (DMSO) every other day from 8th week to 12th week after last STZ injection. Some diabetic mice were injected intraperitoneally with vehicle (10% DMSO) to control for the influence of buffer-induced effects.

OIR model was established as described previously 38-40. Briefly, on P7, mouse pups and the nursing mother were placed in an airtight incubator (own production) ventilated by a mixture of oxygen and air to a final oxygen fraction of 75% ± 2% and age-matched mouse pups in room air as NOIR controls. The oxygen levels were checked at least three times per day. After 5 days of hyperoxia, these mice were returned to room air at P12. For treatment, the mice received intraperitoneal injection (ip.) of 79-6 (50 mg/kg) every other day from P12 to P16 and ones with vehicle (10% DMSO) ip. as controls. At P17, the peak time of retinal angiogenesis, mice were euthanized by carbon dioxide and the retina, blood, spleen were extracted for further analysis.

### Flow cytometry of analysis

Detection of Tfh cells by fluorescence-activated cell sorting (FACS) was performed as described previously 41. Briefly, fresh venous blood from participants (n=10) and mice (NOIR, n=10; OIR, n=12) were collected in heparin sodium anticoagulant tubes and peripheral blood mononuclear cells (PBMCs) were isolated by Ficoll centrifugation. Spleens (n=6) of OIR and diabetic mice were harvested into a culture dish containing 5 mL of cell culture medium and teased apart into a single-cell suspension by pressing with the plunger of a 1-mL syringe. The suspensions were passed through a 70-μm cell strainer to exclude the debris and centrifuged at 300×g for 8 min at 4 °C. The red blood cells were discarded using 1 mL RBC Lysis Buffer. PBMCs and splenocytes were blocked with CD16/CD32 (1:100, Clone 2.4G2, Cat# 553141, BD Biosciences) for 10 min at 4 ℃ after washed with FACS buffer (Phosphate Buffered Saline (PBS) containing 1% FBS and 10 mM Hepes (MP Biomedicals)). Then the cells were incubated with conjugated monoclonal antibodies against CD4 (1:100, Clone RPA-T4, Cat# 555349, Clone RM4-5, Cat# 553051, BD Biosciences), CXCR5 (1:100, Clone RF8B2, Cat# 562747, Clone 2G8, Cat# 560577, BD Biosciences), PD-1 (1:100, Clone MIH4, Cat# 557946, Clone J43, Cat# 551892, BD Biosciences) diluted in FACS buffer at room temperature for 30 min. After washed twice, the samples were analyzed on a four-laser Becton-Dickinson FACS Calibur (BD Biosciences) and the data was analyzed by Flow Jo software.

### Evaluation of retinal vascular permeability by Evans blue

Evans Blue staining was performed to evaluate the permeability of retinal microvessels. Mice received an intraperitoneal injection of Evans blue dye (50 mg/kg). Two hours later, the mice were anesthetized and perfused through the left ventricle with PBS to completely remove the Evans blue dye in blood vessels. Then the mice were sacrificed by carbon dioxide euthanasia and eyes were removed. The eyeballs were fixed in 4% paraformaldehyde (PFA) for 60 min at room temperature. Then retinae were carefully dissected out and cut into four pieces and flat-mounted (n=6 retinae from 3 mice). Vascular morphology and permeability was observed in different locations of the retina (central, mid-peripheral and peripheral areas) using a confocal microscope (Carl Zeiss LSM710, German). The permeability of retinal microvessels was calculated as the percentage of leakage area to total area from four images in the central, mid-peripheral and peripheral retina, respectively.

### Immunofluorescence staining

For cryosections, eyes (n=6 retinae from 6 mice) and cervical lateral LNs (n=6) were removed carefully and embedded in optimal cutting temperature compound (Sakura Fine Technical). The frozen samples were then sliced transversely (8 µm) with a cryostat (Leica CM1950) at -20 °C. Only the cross-sections throughout optic nerve were used for staining and analysis. For retinal flat-mounts, the eyes (n=3 eyes from 3 mice) were fixed in 4% PFA for 60 min at room temperature and the retinae were dissected out as cups. Both cryosections and retinal cups were blocked with PBS containing 0.5% Triton-X100 and 5% BSA at room temperature for 2 h. Cryosections were incubated with primary antibodies at 4 °C overnight and retinal cups were incubated at 4 °C for 2 d. The primary antibodies were diluted in PBS containing 0.5% Triton-X100 and 5% BSA and included anti-PD-1 (1:100, ab214421, Abcam, Cambridge, MA; AF1021, Novus Biologicals), anti-CXCR5 (1:100, ab133706, Abcam, Cambridge, MA), anti-VEGF (1:100, sc-53462, Santa Cruz), anti-FGF2 (1:100, ab8880, Abcam, Cambridge, MA), anti-IL-21 (1:100, ab5978, Abcam, Cambridge, MA) and anti-CD4 antibody (1:100, ab25475, Abcam, Cambridge, CA). After washed with PBST buffer (PBS with 0.1% Tween 20) for three times, the slices and retinal cups were incubated with fluorescently labeled secondary antibodies for 2 h at room temperature. The slices were counterstained with 4'6-diamidino-2-phenylindole (DAPI) for 5 min at room temperature and washed before mounted. After washed with PBST buffer, the retinal cups were cut into four pieces and flat-mounted. The sections and retinal flat-mounts were observed using a confocal microscope (Carl Zeiss LSM710, German).

### IB4 staining for measurement of neovascularization

The IB4 staining of retinal flat-mounts or cryosections was performed as described 24, 38, 42. The retinal cups (OIR + Vehicle, n=10 retinae from 10 mice; OIR + Bcl-6i, n=8 retinae from 8 mice) and cryosections (n=6 retinae from 6 mice) were prepared as above immunofluorescence staining. After blocked, the cups and slices were incubated with IB4 (1:50, Invitrogen, Shanghai, Co. Ltd.) diluted in PBS containing 0.5% Triton-X100 and 5% BSA at room temperature for 2 h. Retinal cups were then washed extensively with PBST buffer and cut into four pieces before flat-mounted. The slices were counterstained with DAPI for 5 min at room temperature and washed before mounted. The images were obtained using a confocal microscope (Carl Zeiss LSM710, German). Neovascular areas on flat-mounts were labeled and quantified using Image-Pro Plus 6.0 (Media Cybernetic, MD, USA) and neovascularization presented as the percentage of neovascular area to the total retinal area. The IB4+ cells were counted from four images/fields of one cryosection, three sections of one eye, and six eyes and calculated as mean positive cells per field.

### Hematoxylin & Eosin (H&E) Staining

H&E staining on retinal sections was performed to characterize the endothelial cells extruding into the vitreous cavity. Eyes were fixed with 4% formalin overnight, embedded in paraffin and cut into 3 μm vertical slices. Sections were washed and treated with hematoxylin buffer for 10 min at room temperature. Then sections were rinsed in deionized water and dipped in 1% Eosin solution for 15 s. After rehydrated in alcohol gradient, slices were washed again and mounted. Histological analyses of retinal tissues (n=6 retinae from 6 mice) were observed under a microscope (Leica DM4000, Germany) for calculating cell nuclei above the internal limiting membrane across the whole retina from 3 sections per retina. Images were processed with ImageJ software (US National Institutes of Health).

### Real time PCR

The mRNA levels of IL-21, CXCL13, VEGF, IL-6, and TNF-α cytokines were detected by real time PCR. The total RNA of retinae (n=6 retinae from 3 mice) were extracted with TRIzol (Invitrogen, Carlsbad, CA, United States) and converted into first-strand cDNA using PrimeScript^TM^ RT reagent Kit (TaKaRa Biotechnology, Co., Ltd., Dalian, China) according to the manufacturer's instructions. qPCR was performed with a total volume of 20 μL containing 2 μL of cDNA, 10 μL of 2×SYBR Premix Ex Taq, and 10 μmol/L of the primer pairs in a Light Cycler 96 (Roche Applied Science, Mannheim, Germany). The primers were listed in the Supplementary Table S2, GAPDH was used as a reference gene. The qPCR amplification protocols consisted of 95 °C for 30 s and up to 40 cycles of 95 °C for 5 s and 60 °C for 34 s according to the manufacturer's instructions.

### Western blotting

The retinae (n=6 retinae from 3 mice) were dissected out and their total protein was extracted and homogenized in lysis buffer (RIPA, Biocolors, Shanghai, China) containing protease and phosphatase inhibitor mini tablets (Thermo Fisher Scientific, no. 88668; Waltham, MA, USA) using TissueLyser (Jingxin Tiss-48, Shanghai, China) at 4 °C. The protein concentration was determined by Bicinchoninic acid (BCA) protein assay and same quantity of protein in each sample was used for the following experiment. Thirty μg protein was loaded per lane on 15% SDS-PAGE gels and transferred to polyvinylidene difluoride membranes (Millipore). The membrane blots were blocked with 5% BSA in PBST for 1 h at room temperature and then incubated with primary antibodies at 4 °C overnight. Primary antibodies included anti-IL-21 antibody (1:500, ab5978, Abcam, Cambridge, MA), anti-VEGF (1:500, sc-53462, Santa Cruz), and anti-β-actin antibodies (1:2000, ab8227, Abcam, Cambridge, MA). After washed with PBST buffer for 3 times, the membrane blots were incubated with horseradish peroxidase (HRP) conjugated secondary antibodies at room temperature for 1 h. Both primary and secondary antibodies were diluted in PBST with 5% BSA. After washed with PBST buffer for 3 times, the membrane blots were detected by the ChemiDoc Imaging System (Bio-Rad, USA). Finally, the gray intensity of proteins was measured using ImageJ software (US National Institutes of Health) to determine the protein concentration.

### Statistical Analysis

All experiments including flow cytometry, Evans blue perfusion, immunostaining, PCR and western blotting were repeated at least 3 times. Neovascularization measurement, cell number calculation and data analysis were performed by two blind researchers. Data are presented as mean ± standard error of the mean (SEM), and were analyzed statistically using one-way ANOVA or independent T-test. The nonparametric tests Mann-Whitney U-test were also used with T-test to give the most accurate estimates of significance. P values < .05 were considered statistically significant.

## Supplementary Material

Supplementary tables.

## Abbreviations

BRB: blood retinal barrier
Bcl-6i: B-cell lymphoma 6 inhibitor
BCA: bicinchoninic acid
DR: diabetic retinopathy
DAPI: 4'6-diamidino-2- phenylindole
FACS: fluorescence-activated cell sorting
GC: germinal center
GCL: ganglion cell layer
IB4: Isolectin B4
ILM: internal limiting membrane
INL: Inner nuclear layer
IPL: inner plexiform layer
LNs: lymph nodes
OIR: oxygen-induced retinopathy
ONL: outer nuclear layer
OPL: outer plexiform layer
PBMCs: peripheral blood mononuclear cells
PBS: Phosphate Buffered Saline
PFA: paraformaldehyde
STZ: streptozotocin
SEM: standard error of the mean
Tfh: follicular helper T

## References

[1] Nentwich MM, Ulbig MW (2015). Diabetic retinopathy - ocular complications of diabetes mellitus. World J Diabetes.

[2] Chen L, Magliano DJ, Zimmet PZ (2011). The worldwide epidemiology of type 2 diabetes mellitus-present and future perspectives. Nat Rev Endocrinol.

[3] Zhang Y, Cai S, Jia Y, Qi C, Sun J, Zhang H (2017). Decoding Noncoding RNAs: Role of MicroRNAs and Long Noncoding RNAs in Ocular Neovascularization. Theranostics.

[4] Ding J, Wong TY (2012). Current epidemiology of diabetic retinopathy and diabetic macular edema. Curr Diab Rep.

[5] Tang J, Kern TS (2011). Inflammation in diabetic retinopathy. Prog Retin Eye Res.

[6] Yu Y, Chen H, Su SB (2015). Neuroinflammatory responses in diabetic retinopathy. J Neuroinflammation.

[7] Yu B, Wang S (2018). Angio-LncRs: LncRNAs that regulate angiogenesis and vascular disease. Theranostics.

[8] El-Asrar AM, Nawaz MI, Kangave D, Geboes K, Ola MS, Ahmad S (2011). High-mobility group box-1 and biomarkers of inflammation in the vitreous from patients with proliferative diabetic retinopathy. Mol Vis.

[9] Urbancic M, Stunf S, Milutinovic Zivin A, Petrovic D, GlobocnikPetrovic M (2014). Epiretinal membrane inflammatory cell density might reflect the activity of proliferative diabetic retinopathy. Invest Ophthalmol Vis Sci.

[10] Lintermans LL, Stegeman CA, Heeringa P, Abdulahad WH (2014). T cells in vascular inflammatory diseases. Front Immunol.

[11] Takeuchi M, Sato T, Tanaka A, Muraoka T, Taguchi M, Sakurai Y (2015). Elevated Levels of Cytokines Associated with Th2 and Th17 Cells in Vitreous Fluid of Proliferative Diabetic Retinopathy Patients. PLoS One.

[12] Canton A, Martinez-Caceres EM, Hernandez C, Espejo C, Garcia-Arumi J, Simo R (2004). CD4-CD8 and CD28 expression in T cells infiltrating the vitreous fluid in patients with proliferative diabetic retinopathy: a flow cytometric analysis. Arch Ophthalmol.

[13] Han XQ, Gong ZJ, Xu SQ, Li X, Wang LK, Wu SM (2014). Advanced glycation end products promote differentiation of CD4(+) T helper cells toward pro-inflammatory response. J Huazhong Univ Sci Technolog Med Sci.

[14] Ilan Y, Maron R, Tukpah AM, Maioli TU, Murugaiyan G, Yang K (2010). Induction of regulatory T cells decreases adipose inflammation and alleviates insulin resistance in ob/ob mice. Proc Natl Acad Sci U S A.

[15] Crotty S (2014). T follicular helper cell differentiation, function, and roles in disease. Immunity.

[16] Tangye SG, Ma CS, Brink R, Deenick EK (2013). The good, the bad and the ugly - TFH cells in human health and disease. Nat Rev Immunol.

[17] Craft JE (2012). Follicular helper T cells in immunity and systemic autoimmunity. Nat Rev Rheumatol.

[18] Heuts F, Edner NM, Walker LS (2017). Follicular T Helper Cells: A New Marker of Type 1 Diabetes Risk?. Diabetes.

[19] Kenefeck R, Wang CJ, Kapadi T, Wardzinski L, Attridge K, Clough LE (2015). Follicular helper T cell signature in type 1 diabetes. J Clin Invest.

[20] Schmitt N, Bentebibel SE, Ueno H (2014). Phenotype and functions of memory Tfh cells in human blood. Trends Immunol.

[21] Le Coz C, Joublin A, Pasquali JL, Korganow AS, Dumortier H, Monneaux F (2013). Circulating TFH subset distribution is strongly affected in lupus patients with an active disease. PLoS One.

[22] Miyamoto K, Khosrof S, Bursell SE, Rohan R, Murata T, Clermont AC (1999). Prevention of leukostasis and vascular leakage in streptozotocin-induced diabetic retinopathy via intercellular adhesion molecule-1 inhibition. Proc Natl Acad Sci U S A.

[23] Robinson R, Barathi VA, Chaurasia SS, Wong TY, Kern TS (2012). Update on animal models of diabetic retinopathy: from molecular approaches to mice and higher mammals. Dis Model Mech.

[24] He C, Lai P, Wang J, Zhou T, Huang Z, Zhou L (2016). TLR2/4 deficiency prevents oxygen-induced vascular degeneration and promotes revascularization by downregulating IL-17 in the retina. Sci Rep.

[25] Nurieva RI, Chung Y, Martinez GJ, Yang XO, Tanaka S, Matskevitch TD (2009). Bcl6 mediates the development of T follicular helper cells. Science.

[26] Yuan MJ, Wang T (2016). Advances of the interleukin-21 signaling pathway in immunity and angiogenesis. Biomed Rep.

[27] Viisanen T, Ihantola EL, Nanto-Salonen K, Hyoty H, Nurminen N, Selvenius J (2017). Circulating CXCR5+PD-1+ICOS+ Follicular T Helper Cells Are Increased Close to the Diagnosis of Type 1 Diabetes in Children With Multiple Autoantibodies. Diabetes.

[28] Peters A, Pitcher LA, Sullivan JM, Mitsdoerffer M, Acton SE, Franz B (2011). Th17 cells induce ectopic lymphoid follicles in central nervous system tissue inflammation. Immunity.

[29] Carragher DM, Rangel-Moreno J, Randall TD (2008). Ectopic lymphoid tissues and local immunity. Semin Immunol.

[30] King C, Tangye SG, Mackay CR (2008). T follicular helper (TFH) cells in normal and dysregulated immune responses. Annu Rev Immunol.

[31] Moser B, Schaerli P, Loetscher P (2002). CXCR5(+) T cells: follicular homing takes center stage in T-helper-cell responses. Trends Immunol.

[32] Zhang X, Ing S, Fraser A, Chen M, Khan O, Zakem J (2013). Follicular helper T cells: new insights into mechanisms of autoimmune diseases. Ochsner J.

[33] Liu SM, King C (2013). IL-21-producing Th cells in immunity and autoimmunity. J Immunol.

[34] Ma CS, Deenick EK (2014). Human T follicular helper (Tfh) cells and disease. Immunol Cell Biol.

[35] Hatzi K, Nance JP, Kroenke MA, Bothwell M, Haddad EK, Melnick A (2015). BCL6 orchestrates Tfh cell differentiation via multiple distinct mechanisms. J Exp Med.

[36] Ma CS, Deenick EK, Batten M, Tangye SG (2012). The origins, function, and regulation of T follicular helper cells. J Exp Med.

[37] Cerchietti LC, Ghetu AF, Zhu X, Da Silva GF, Zhong S, Matthews M (2010). A small-molecule inhibitor of BCL6 kills DLBCL cells in vitro and in vivo. Cancer Cell.

[38] He C, Sun Y, Ren X, Lin Q, Hu X, Huang X (2013). Angiogenesis mediated by toll-like receptor 4 in ischemic neural tissue. Arterioscler Thromb Vasc Biol.

[39] Liu C, Yao MD, Li CP, Shan K, Yang H, Wang JJ (2017). Silencing Of Circular RNA-ZNF609 Ameliorates Vascular Endothelial Dysfunction. Theranostics.

[40] Sun J, Huang W, Yang SF, Zhang XP, Yu Q, Zhang ZQ (2018). Galphai1 and Galphai3mediate VEGF-induced VEGFR2 endocytosis, signaling and angiogenesis. Theranostics.

[41] Dambuza IM, He C, Choi JK, Yu CR, Wang R, Mattapallil MJ (2017). IL-12p35 induces expansion of IL-10 and IL-35-expressing regulatory B cells and ameliorates autoimmune disease. Nat Commun.

[42] He C, Zhao C, Kumar A, Lee C, Chen M, Huang L (2014). Vasoprotective effect of PDGF-CC mediated by HMOX1 rescues retinal degeneration. Proc Natl Acad Sci U S A.

