# Supplementary material for: Bcl-6-directed follicular helper T cells promote vascular inflammatory injury in diabetic retinopathy
Source: Theranostics. 2020 Mar 4;10(9):4250–64. doi: 10.7150/thno.43731 (PMC7086358; doi:10.7150/thno.43731)
Supplement: Supplementary file 1 — Supplementary tables. [file thnov10p4250s1.pdf]

Table S1 Characteristics of patients with DR.

Characteristic	DR Patients	Controls
Patients	10	10
Clinical features		
Male/female	5/5	6/4
Age (years)	56.8 ± 7.3	58.6 ± 8.9
Type 1/type 2 diabetes	2/8	0
Duration of diabetes (years)	4.8 ± 2.7	0
Insulin treatment	3	0
Hypertension	3	4
Dyslipidaemia	3	3
Retinal features		
Microaneurysm	7	0
Hard exudates	6	0
Cotton-wool spots	4	0
Retinal haemorrhage	6	0
Retinal angiogenesis	5	0
Vitreous haemorrhage	3	0
Macular oedema	2	0
Therapies		
Intravitreal injection of anti-VEGF agents	9	0
Pan-retinal laser photocoagulation	2	0

Table S2. Primers sequence for real time PCR

Gene name	Orientation	Primer sequence (5’ to 3’)	Species
Il21	Forward	GGGGACAGTGGCCCATAAATC	Mouse
	Reverse	GTGCCCCTTTACATCTTGTGG	Mouse
Cxcl13	Forward	ATATGTGTGAATCCTCGTGCCA	Mouse
	Reverse	GGGAGTTGAAGACAGACTTTTGC	Mouse
vegf	Forward	CTGCCGTCCGATTGAGACC	Mouse
	Reverse	CCCCTCCTTGTACCACTGTC	Mouse
il-6	Forward	TCTATACCACTTCACAAGTCGGA	Mouse
	Reverse	GAATTGCCATTGCACAACTCTTT	Mouse
tnf-α	Forward	CAGGCGGTGCCTATGTCTC	Mouse
	Reverse	CGATCACCCCGAAGTTCAGTAGTAA	Mouse
Cxcr5	Forward	TGGCCTTCTACAGTAACAGCA	Mouse
	Reverse	GCATGAATACCGCCTTAAAGGAC	Mouse
Bcl-6	Forward	CCGGCACGCTAGTGATGTT	Mouse
	Reverse	GCACTGTCTTATGGGCTCTAAAC	Mouse
gapdh	Forward	GCCAAGGCTGTGGGCAAGGT	Mouse
	Reverse	TCTCCAGGCGGCACGTCAGA	Mouse
